# Cortical circuits for silent speechreading in deaf and hearing people

**DOI:** 10.1016/j.neuropsychologia.2007.11.026

**Published:** 2008

**Authors:** Cheryl M. Capek, Mairéad MacSweeney, Bencie Woll, Dafydd Waters, Philip K. McGuire, Anthony S. David, Michael J. Brammer, Ruth Campbell

**Affiliations:** a Deafness, Cognition and Language Research Centre, Division of Psychology and Language Sciences, University College London, 49 Gordon Square, London WC1H 0PD, United Kingdom; b Behavioural and Brain Sciences Unit, UCL Institute of Child Health, University College London, 30 Guilford Street, London WC1N 1EH, United Kingdom; c Institute of Psychiatry, Kings College London, De Crespigny Park, London SE5 8AF, United Kingdom

**Keywords:** Deafness, Brain, Language, Sign language, Speechreading, fMRI

## Abstract

This fMRI study explored the functional neural organisation of seen speech in congenitally deaf native signers and hearing non-signers. Both groups showed extensive activation in perisylvian regions for speechreading words compared to viewing the model at rest. In contrast to earlier findings, activation in left middle and posterior portions of superior temporal cortex, including regions within the lateral sulcus and the superior and middle temporal gyri, was greater for deaf than hearing participants. This activation pattern survived covarying for speechreading skill, which was better in deaf than hearing participants. Furthermore, correlational analysis showed that regions of activation related to speechreading skill varied with the hearing status of the observers. Deaf participants showed a positive correlation between speechreading skill and activation in the middle/posterior superior temporal cortex. In hearing participants, however, more posterior and inferior temporal activation (including fusiform and lingual gyri) was positively correlated with speechreading skill. Together, these findings indicate that activation in the left superior temporal regions for silent speechreading can be modulated by both hearing status and speechreading skill.

## 1 Introduction

Auditory speech processing reliably engages perisylvian regions, particularly in the left hemisphere (e.g., Scott & Johnsrude, 2003). In hearing people, perisylvian regions are also recruited for silent speechreading. In particular, silent speechreading elicits activation in superior temporal regions, including middle and posterior portions of the superior temporal gyrus, its dorsal and ventral surfaces (i.e., lateral sulcus and superior temporal sulcus or STS, respectively) and the middle temporal gyrus (Bernstein et al., 2002; Calvert et al., 1997; Calvert et al., 1999; Calvert, Campbell, & Brammer, 2000; Ludman et al., 2000; MacSweeney et al., 2000; Paulesu et al., 2003; Pekkola et al., 2005; Ruytjens, Albers, van Dijk, Wit, & Willemsen, 2006), and inferior frontal regions (Buccino et al., 2004; Campbell et al., 2001; Nishitani & Hari, 2002; Paulesu et al., 2003; Watkins, Strafella, & Paus, 2003). Generally, seen speech appears to engage similar circuits to those activated when speech is heard. This includes portions of the superior temporal cortex reliably involved in processing auditory information. Activation in this region also appears to be modulated by speechreading skill. Hall, Fussell, and Summerfield (2005) did not find marked activation in the superior temporal gyrus at the group level when hearing adults observed silently spoken sentences, as compared to viewing facial gurning. However, their participants varied greatly in their ability to speechread, and a positive correlation was found between activation in the left posterior superior temporal gyrus and speechreading skill.

Deaf people can outperform hearing people in comprehending seen speech (Bernstein, Demorest, & Tucker, 2000; Mohammed, Campbell, MacSweeney, Barry, & Coleman, 2006). Nevertheless, earlier reports suggested that superior temporal activation for speechreading was less reliably observed in deaf than in hearing people (MacSweeney et al., 2001; MacSweeney et al., 2002). However, the group size for these studies was small (*n* = 6), and so there may not have been sufficient statistical power to detect activation in this region. Furthermore, while the speechreading task in MacSweeney et al.'s (2002) study was easy (identify spoken numbers between 1 and 9), it was compared with a relatively high-level task—counting numbers of meaningless mouth actions. In contrast, a separate study by Sadato et al. (2005), reported activation in superior temporal regions in both hearing and deaf participants viewing speech-like actions. Here, the stimulus was a cartoon avatar opening and closing its mouth to form different vowel-like patterns, which participants may have interpreted as phonological gestures.

The present study is the first to examine patterns of activation in deaf people who are proficient speechreaders while they searched for a speechread target embedded in lists of unrelated words. We anticipated that both hearing status and speechreading ability, measured outside the scanner, may determine the extent of activation in perisylvian regions. This was explored in two complementary ways. First, the group comparison between deaf and hearing activation patterns was assessed with speechreading skill entered into the analysis as a covariate. Speechreading skill was assessed using the Test of Adult Speechreading (TAS, Mohammed et al., 2006). By ‘partialling out’ individual differences in speechreading ability, we hoped to establish whether activation in brain regions could be modulated as a function of hearing status, irrespective of speechreading skill. Second, we used correlational analysis to establish, for each group in turn, which regions were sensitive to variations in speechreading skill.

To summarise, this study examines cortical correlates for the perception of lists of speechread words under lexical target detection conditions. We aimed to identify regions that may be activated during observation of silently spoken lexical items that are not drawn from a closed set, and when the contrast (baseline) condition was a speaker at rest. The questions posed were: (1) To what extent do prelingually deaf people who are proficient signers and speechreaders show activation in superior temporal regions, including auditory cortical processing regions? (2) Are the patterns of activation different in deaf and hearing people? (3) In which regions is speechreading ability positively correlated with activation?

## 2 Method

### 2.1 Participants

Thirteen (six female; mean age: 27.4; age range: 18–49) deaf adults were tested. All were congenitally (severely or profoundly) deaf (81 dB mean loss or greater in the better ear over four octaves, spanning 500–4000 Hz). Across the group, the mean hearing loss in the better ear was 103 dB. They were all native signers, having acquired British Sign Language (BSL) from their deaf signing parents. Thirteen (six female; mean age: 29.4; age range: 18–43) hearing, monolingual speakers of English were also tested. All participants were right-handed with no known neurological or behavioural abnormalities. Non-verbal IQ was measured using the Block Design subtest of the WAIS-R. Speechreading was measured using the Test of Adult Speechreading (TAS). The TAS comprises three subtests of silent speechreading in English: word identification, sentence identification, and short story identification (Mohammed et al., 2006). Independent-samples *t*-tests showed that deaf and hearing participants did not differ on non-verbal IQ (*p* > 0.1). However, deaf participants scored significantly higher than hearing non-signers on the TAS (*t* (24) = 4.779, *p* < 0.001), confirming earlier findings (Mohammed et al., 2006) with an independent sample of participants. Participant characteristics are summarised in Table 1. Standard scores (TAS *z*-scores) were derived from the populations reported in Mohammed et al.'s (2006) study, together with those for the present study. These scores were calculated separately for deaf and for hearing groups. Standard scores were used in order to correct for the differences in statistical distribution of scores within the deaf and the hearing groups, and were used in the correlational analyses (see Table 1).

All participants gave written informed consent to participate in the study according to the Declaration of Helsinki (BMJ 1991; 302: 1194) and the study was approved by the Institute of Psychiatry/South London and Maudsley NHS Trust Research Ethics Committee.

### 2.2 Stimuli

Stimuli were full-colour motion video of silently mouthed English words. Stimuli were modelled by a deaf native signer of BSL, who spoke English fluently (i.e., a BSL-English bilingual). The model was viewed full-face and torso. The words to be speechread were piloted on adult hearing volunteers who were not scanned. The final stimuli comprised only those words that were speechreadable by the hearing pilots. Stimuli consisted of both content words (nouns) and descriptive terms (both adjectival and adverbial).

### 2.3 fMRI experimental design and task

The speechreading task was one of four conditions presented to participants. The other three conditions comprised signed language (BSL) material (not reported here). The speech stimuli were presented in blocks, alternating with blocks of the other three experimental conditions (30-s blocks for each condition), and with a 15-s baseline condition. The total run duration for all four conditions and baseline was 15 min. Both deaf and hearing participants were given the same target-detection task and instructions. During the speechreading condition, participants were instructed to watch the speech patterns produced by the model and to try to understand them. They were required to make a push-button response whenever the model was seen to be saying ‘yes’. This relatively passive task was chosen in preference to a ‘deeper’ processing task (such as semantic classification) for several reasons. First, it allowed for relatively automatic processing of non-target items to occur (as confirmed in post-scan tests). Second, it ensured similar difficulty of the task across stimulus conditions. As hearing non-signers would not be able to perform a semantic task on the sign stimuli, using a sparse target detection task enabled all participants to perform the same task during all experimental conditions. Over the course of the experiment, participants viewed 96 stimulus items, 24 in each of the four experimental conditions. Items were not repeated within the same block and were pseudorandomised to ensure that repeats were not clustered at the end of the experiment. Each participant saw five blocks of the speechreading condition.

The baseline condition comprised video of the model at rest. The model's face and torso were shown, as in the experimental conditions. During the baseline condition, participants were directed to press a button when a grey fixation cross, digitally superimposed on the face region of the resting model, turned red. To maintain vigilance, targets in both the experimental and baseline conditions occurred randomly at a rate of one per block. Prior to the scan, participants practiced the tasks and were shown examples of the ‘yes’ targets outside the scanner using video of a model and words that were similar but not identical to those used in the experiment. Following the experiment, a sample of the hearing participants (8 of 13) and all of the deaf participants were asked to identify the items they had seen.

Stimuli in the experimental conditions appeared at a rate of 15 items per block. The rate of articulation across all experimental conditions, including the speechreading blocks, was approximately one item every 2 s. All stimuli were projected onto a screen located at the base of the scanner table via a Sanyo XU40 LCD projector and then projected to a mirror angled above the participant's head.

### 2.4 Imaging parameters

Gradient echoplanar MRI data were acquired with a 1.5-T General Electric Signa Excite (Milwaukee, WI, USA) with TwinSpeed gradients and fitted with an 8-channel quadrature head coil. Three hundred $T_{2}^{*}$-weighted images depicting BOLD contrast were acquired at each of the 40 near-axial 3 mm thick planes parallel to the intercommissural (AC-PC) line (0.3 mm interslice gap; TR = 3 s, TE = 40 ms, flip angle = 90°). The field of view for the fMRI runs was 240 mm, and the matrix size was 64 × 64, with a resultant in-plane voxel size of 3.75 mm. High-resolution EPI scans were acquired to facilitate registration of individual fMRI datasets to Talairach space (Talairach & Tournoux, 1988). These comprised 40 near-axial 3 mm slices (0.3 mm gap), which were acquired parallel to the AC-PC line. The field of view for these scans was matched to that of the fMRI scans, but the matrix size was increased to 128 × 128, resulting in an in-plane voxel size of 1.875 mm. Other scan parameters (TR = 3 s, TE = 40 ms, flip angle = 90°) were, where possible, matched to those of the main EPI run, resulting in similar image contrast.

### 2.5 Data analysis

The fMRI data were first corrected for motion artefact, then smoothed using a Gaussian filter (FWHM 7.2 mm) to improve the signal to noise ratio over each voxel and its immediate neighbours prior to data analysis. In addition, low frequency trends were removed by a wavelet-based procedure in which the time series at each voxel was first transformed into the wavelet domain and the wavelet coefficients of the three levels corresponding to the lowest temporal frequencies of the data were set to zero. The wavelet transform was then inverted to give the detrended time series. The least-squares fit was computed between the observed time series at each voxel and the convolutions of two gamma variate functions (peak responses at 4 and 8 s) with the experimental design (Friston, Josephs, Rees, & Turner, 1998). The best fit between the weighted sum of these convolutions and the time series at each voxel was computed using the constrained BOLD effect model suggested by Friman, Borga, Lundberg, and Knutsson (2003) in order to constrain the range of fits to those that reflect the physiological features of the BOLD response^1^.

Following computation of the model fit, a goodness of fit statistic was derived by calculating the ratio between the sum of squares due to the model fit and the residual sum of squares (SSQ ratio) at each voxel. Permutation testing, as well as its freedom from many of the distributional assumptions of parametric tests, also offers the possibility of testing a number of statistics that are not easily testable parametrically. The SSQ ratio is such a statistic and is a simplified substitute for the *F* statistic suggested by Edgington (1995) that avoids the necessity of calculating the residual degrees of freedom of the time series following model fitting.

The data were permuted by the wavelet-based method described by Bullmore et al. (2001) with the exception that, prior to permutation, any wavelet coefficients exceeding the calculated threshold (as described by Donoho and Johnstone (1994)) were removed. These were replaced by the threshold value. This step reduces the likelihood of refitting large, experimentally unrelated components of the signal following permutation.

Significant values of the SSQ were identified by comparing this statistic with the null distribution, determined by repeating the fitting procedure 20 times at each voxel and combining data over all intracerebral voxels. This procedure preserves the noise characteristics of the time series during the permutation process, and the global assessment of the null distribution performed in this way provides good control of Type I error rates (Bullmore et al., 2001). The voxel-wise SSQ ratios were calculated for each subject from the observed data and, following time series permutation, were transformed into standard space (Talairach & Tournoux, 1988) as described previously (Brammer et al., 1997; Bullmore et al., 1996). The Talairach transformation stage was performed in two parts. First, each participant's fMRI data was realigned with their own high resolution $T_{2}^{*}$-weighted images using a rigid body transformation. Second, an affine transformation to the Talairach template was computed. The cost function for both transformations was the maximization of the correlation between the images. Voxel size in Talairach space was 3 mm × 3 mm × 3 mm.

### 2.6 Group analysis

Identification of active 3-D clusters was performed by first thresholding the median voxel-level SSQ ratio maps at the false positive probability of 0.05. The activated voxels were assembled into 3-D connected clusters and the sum of the SSQ ratios (statistical cluster mass) was determined for each cluster. This procedure was repeated for the median SSQ ratio maps obtained from the wavelet-permuted data to compute the null distribution of statistical cluster masses under the null hypothesis. The cluster-wise false positive threshold was then set using this distribution to give an expected false positive rate of <1 cluster per brain (Bullmore et al., 1999).

### 2.7 ANOVA

Differences between the groups were calculated by fitting the data at each voxel in which all participants had non-zero data using the following linear model, *Y* = *a* + *bX* + *e*, where *Y* is the vector of BOLD effect sizes for each individual, *X* is the contrast matrix for the particular inter-group contrast required, *a* is the mean effect across all individuals in the groups, *b* is the computed group difference and *e* is a vector of residual errors. The model is fitted by minimising the sum of absolute deviations rather than the sums of squares to reduce outlier effects. The null distribution of *b* is computed by permuting data between groups (assuming the null hypothesis of no effect of group) and refitting the above model. This permutation method thus gives an exact test (for this set of data) of the probability of the value of *b* in the unpermuted data under the null hypothesis. The permutation process permits estimation of the distribution of *b* under the null hypothesis of no mean difference. Identification of significantly activated clusters was performed by using the cluster-wise false positive threshold that yielded an expected false positive rate of <1 cluster per brain (Bullmore et al., 1999).

### 2.8 ANCOVA

Analysis of covariance was used to address behavioural differences between the deaf and hearing participants in relation to the patterns of activation for the speechreading condition (see Table 1). Differences in responses (*R*) were inferred at each voxel using the linear model, *R* = *a*0 + *a*1*H* + *a*2*X* + *e*, where *H* codes the contrast(s) of interest between groups, *X* is a covariate and *e* is the residual error. Maps of the standardized coefficient (size of group difference) (*a*1), were tested for significance against the null distribution of *a*1 (no effect of group membership) generated by repeatedly refitting the above model at each voxel following randomization of group membership (*H*).

### 2.9 Correlational analysis

In order to examine the relationship between brain activation and speechreading skill, correlational analysis was performed between the BOLD effect data for each individual and the Test of Adult Speechreading (TAS) *z*-score. These were calculated separately for each group. Pearson product–moment correlation coefficients were calculated between the observed behavioural and BOLD effect data. The null distribution of correlation coefficients was then computed by permuting the BOLD data 100 times per voxel and then combining the data over all voxels. Median voxel-level maps were computed at the false probability of 0.05 and cluster-level maps, where *r* was significant, were computed such that the expected false positive rate was <1 cluster per brain.

## 3 Results

### 3.1 Behavioural data

All participants completed the behavioural (target detection) task in the scanner reasonably accurately. Deaf participants identified the speechreading targets more accurately than hearing participants (mean accuracy (max = 5), deaf = 4.69, hearing = 3.85, *t*(24) = 2.99, *p* = 0.007). Speechreading target identification was slower in deaf than hearing participants (mean RT, deaf = 1192.63 ms, hearing = 920.08 ms, *t*(24) = 4.15, *p* < 0.001). Following scanning, participants were presented with the experimental stimuli. The deaf participants identified more words than the hearing participants (mean percent correct identification, deaf = 69%, hearing = 46%), *t*(19) = 4.11, *p* = 0.001). The behavioural data suggest that deaf participants’ greater accuracy in identification of non-target items (as indicated by the post-scan test) may have interfered with their processing of the target (as indicated by the relatively slow reaction times to targets in the scanner).

### 3.2 fMRI data

#### 3.2.1 Speechreading vs. baseline

**Fig. 1:**
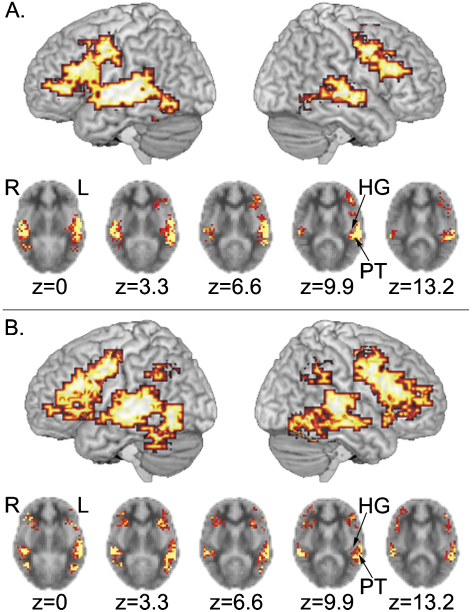
Activation for speechreading relative to the baseline task for each group. (A) Deaf group (top) and (B) hearing group (bottom); 13 participants per group; voxel-wise *p*-value = 0.05, cluster-wise *p*-value = 0.0025. Activations on lateral renderings are displayed up to 15 mm beneath the cortical surface. Five sequential axial sections, showing activation in superior temporal regions, including the planum temporale (PT) and Heschl's gyrus (HG) are also displayed.

In both deaf and hearing groups, extensive activation was observed in fronto-temporal cortices, bilaterally (Table 2, Fig. 1). In deaf participants, activation in the left superior temporal cortex was focused at the border between the posterior superior temporal gyrus and the transverse temporal gyrus (BA 42/41) and extended to the middle (BA 21) and inferior (BAs 37, 19) temporal gyri and the supramarginal gyrus (BA 40). This cluster of activation also extended to inferior (BAs 44, 45) and middle (BAs 6, 9) frontal gyri and precentral gyrus (BA 4). In the right hemisphere, a cluster of activation focused in the superior/middle temporal gyri (BA 22/21) extended to BAs 42 and 41 and posterior inferior temporal gyrus (BAs 37, 19). Activation in the right frontal cortex was focused in the precentral gyrus (BA 6) and extended to the inferior (BAs 44, 45) and middle (BAs 46, 9) frontal gyri. Additional activation was observed at the border of the medial frontal gyrus and the anterior cingulate gyrus (BA 6/32).

In hearing participants, we observed activation focused in the left middle temporo-occipital junction (BA 37) and in the right superior/middle temporal gyrus (BA 22/21). These clusters of activation extended to include the superior and transverse temporal gyri (BAs 22, 42, 41), the postcentral gyri (BA 43) and the middle and inferior temporal (BAs 21, 37, 19, 20) and cerebellar gyri. In the left hemisphere, this cluster also extended to the supramarginal gyrus (BA 40). In both hemispheres, clusters in the inferior parietal cortex were focused in the supramarginal gyrus (BA 40). These clusters extended to angular (BA 39) and middle occipital (BA 19) gyri. The cluster in the right hemisphere extended medially to the border of the dorsal posterior cingulate gyrus (BA 31). Activation in frontal cortices was focused in the precentral gyrus (BA 4/6) of the left hemisphere and in the inferior frontal gyrus (BA 44) of the right hemisphere. In both hemispheres, frontal activation included the inferior (BAs 44, 45 47) middle (BA 46) and superior (BA 9) frontal gyri and the precentral gyrus (BAs 4, 6). In the right hemisphere, the frontal cluster extended anteriorly to the border of the frontal pole (BA 10). Additional activation was observed in the right medial frontal gyrus (BA 6), extending to medial BA 8 and anterior cingulate gyrus (BAs 24 and 32).^2^

#### 3.2.2 Deaf vs. hearing

Deaf native signers displayed significantly greater activation in left and right superior temporal cortices than hearing non-signers. In the left hemisphere, the cluster of activation (116 voxels) was focused at the border between the posterior superior temporal gyrus (i.e., planum temporale) and the transverse temporal (i.e., Heschl's) gyrus (BA 42/41; *x* = −54, *y* = −22, *z* = 10). In the right hemisphere, the cluster (61 voxels) was focused at the border between the superior and middle temporal gyri (BA 22/21; *x* = 51 *y* = −7 *z* = −3). Hearing non-signers showed greater activation than deaf signers in the right prefrontal cortex (128 voxels, focused in BA 44; *x* = 40, *y* = 11, *z* = 26).

**Fig. 2:**
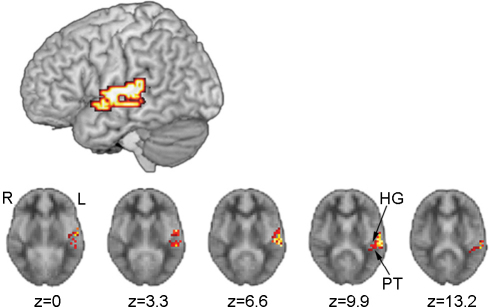
Activation for the speechreading condition as a function of hearing status. The region indicated was more active for deaf than hearing participants, when performance on the TAS (*z*-score) was entered as a covariate (voxel-wise *p*-value = 0.05, cluster-wise *p*-value = 0.01). No regions were more active for hearing than deaf participants. No right hemisphere regions were significantly different across the groups. Five sequential axial sections, showing activation in superior temporal regions, including the planum temporale (PT) and Heschl's gyrus (HG) are also displayed.

When speechreading performance, as indicated by individual TAS *z*-score, was entered as a covariate into this analysis, deaf participants displayed greater activation than hearing participants in the left temporal cortex. The cluster of activation (120 voxels) was focused at the border between the posterior superior temporal gyrus (i.e., planum temporale) and the transverse temporal (i.e., Heschl's) gyrus (BA 42/41; *x* = −54, *y* = −22, *z* = 10). The focus of this cluster was verified using probabilistic maps provided by Penhune, Zatorre, MacDonald, and Evans (1996) (25–50% probability of Heschl's gyrus) and Westbury, Zatorre, and Evans (1999) (26–45% probability of planum temporale). Based on these probability maps, 15 voxels within this cluster, displayed ≥50% probability of being located in Heschl's gyrus, and five voxels showed ≥46% probability of being in planum temporale. This cluster also extended into the posterior lateral portion of the superior temporal gyrus (BA 22) and the middle and posterior portions of the superior temporal sulcus and middle temporal gyrus (BA 21; see Fig. 2). No brain regions were significantly more active in hearing than deaf participants when speechreading was a covariate in the analysis.

#### 3.2.3 Cortical activation for speechreading: correlations with speechreading skill

Speechreading skill, as measured by performance on the Test of Adult Speechreading (TAS), varied considerably across participants (Table 1). Several brain regions were significantly positively associated with TAS *z*-scores in both deaf and hearing groups.

#### 3.2.4 Deaf group

In the deaf group, ten clusters of activation (≥5 voxels) were positively associated with speechreading skill (see Table 3). In the temporal lobe, clusters in the superior temporal cortex were focused in the lateral portion of the transverse temporal gyrus (BA 41) in the right hemisphere, and in the superior temporal gyrus (BA 42) in the left hemisphere. However, although the Talairach and Tournoux (1988) atlas suggests that this cluster incorporates the transverse temporal gyrus, the probability map of this region provided by Penhune et al. (1996) suggests otherwise. In fact, only one voxel (in the left hemisphere cluster) displayed a ≥50% probability of being located in this region (Penhune et al., 1996). Both clusters extended to include the posterior superior temporal gyrus (BAs 42, 22). Additional areas showing significant correlation included the middle portion of the right middle temporal gyrus (BA 21). In the frontal cortex, correlations were observed in the middle frontal gyri of both hemispheres (BA 6). In the right hemisphere, correlations were also observed in the dorsolateral prefrontal cortex (BA 46), precentral gyrus (BA 6/4) and in the anterior insula. Additional correlations were observed in the anterior cingulate gyrus (BA 32/24) and the cerebellum.

#### 3.2.5 Hearing group

In the hearing group, clusters of activation that were positively correlated with TAS *z*-scores included the fusiform (BA 37) and lingual (BA 18) gyri of the right hemisphere and the right postcentral gyrus (BA 4). Additional positive correlations were observed in the posterior cingulate gyrus (BA 23).

## 4 Discussion

Deaf participants were better speechreaders than hearing participants, both in terms of their TAS performance (Table 1) and, when tested post-scan at identifying the words presented in the scanner. The finding that deaf people can be better speechreaders than hearing individuals is not new (Bernstein et al., 2000; Mohammed et al., 2006). Deaf people, including deaf people who use a signed language, rely on speechreading, whether hearing-aid supported or un-aided, to communicate in the wider hearing community. In contrast, in hearing people, where the auditory channel dominates for speech identification, reliance on silent seen speech is generally unfamiliar and unpractised. In the present study most participants, whether deaf or hearing, could speechread much of the spoken material, and it can be assumed, therefore, that some of what they were shown in the scanner was lexically processed—albeit more in deaf than in hearing participants. Interpretation of the imaging data must bear these considerations in mind. Covariance and correlational analyses allow the behavioural and neuroimaging results to be aligned.

The group-level analyses, conducted separately for the deaf and hearing groups, contrasted silent speechreading with a low-level target detection task. As such, these analyses cannot allow unambiguous interpretation of the specificity of such activation in relation to speechreading alone, but they do suggest a general pattern against which the group differences can be explored. In hearing people, the pattern of activation replicates that which has been observed in many previous studies, showing extensive activation across the temporal cortex. While some of this activation must relate to visual movement detection and to the perception of biological motion, especially in posterior and inferior regions (see, for example, Zeki et al., 1991), it is likely that much of the activation in superior temporal regions relates to speechreading, since several studies contrasting speechreading with a higher-level baseline, such as observing non-speech-like mouth movements, report enhanced activation in this region (e.g., Calvert et al., 1997; Paulesu et al., 2003). The present study found that, in both hearing and deaf participants, activation associated with speechreading words included the dorsal surface of the superior temporal cortex including the junction of the superior temporal gyrus and the lateral portion of the transverse temporal (Heschl's) gyrus (BA 42/41). Spatial smoothing intrinsic to transforming data into standard brain space may limit the spatial resolution in this study. Thus the finding that activation for silent speechreading included the lateral portion of Heschl's gyrus must be interpreted with caution. Nevertheless, this finding is consistent with previous neuroimaging research that delineated this region on individual brains (Pekkola et al., 2005). In addition, left inferior frontal regions were activated when observing speech silently. This has also been observed where the contrasts were with higher-level conditions such as watching non-vocal mouth actions (Buccino et al., 2004; Campbell et al., 2001; Paulesu et al., 2003; Watkins et al., 2003) and may reflect the operation of mirror neuron systems in the observation of speech actions.

The finding of superior temporal activation for speechreading in deaf people extends earlier studies exploring the neural organisation of processing a variety of oral gestures in hearing people. This pattern of superior temporal activation found in the present study is consistent with the findings recently reported by Sadato et al. (2005), who presented deaf participants with simple segmental utterances including vowel-like lip shapes. At first sight, the present results do not fit with those we have previously reported using a closed stimulus set, covert articulation and a gurning control condition conducted with a small group of deaf people (MacSweeney et al., 2001; MacSweeney et al., 2002). However, we did report activation within right superior temporal regions, when analysis combining the data from two experiments allowed for an increase in power (MacSweeney et al., 2002). A further study involving a larger group of deaf participants, and manipulating task, baseline condition and stimuli, will help establish whether our previous studies simply lacked power or whether task and stimulus factors systematically affect the extent to which superior temporal regions are recruited during silent speechreading in those born profoundly deaf.

### 4.1 Deaf vs. hearing

When hearing non-signers were compared with deaf signers, and speechreading skill (which differed between the groups) was entered as a covariate (Fig. 2) greater activation was observed for the deaf than hearing group in left middle-posterior superior temporal regions. This cluster of activation was focused at the border between the posterior and transverse temporal gyri (BA 42/41) and extended to the middle and posterior portions of the superior temporal gyrus and sulcus, and middle temporal gyrus. No regions showed greater activation in hearing than deaf participants. In hearing people, the role of the posterior superior temporal sulcus (p-STS) has been proposed as a key ‘binding site’, responsible for cross- and supra-modal processing of co-incident auditory and visual streams in audiovisual speech processing (Calvert et al., 1999; Calvert et al., 2000). However, in deaf people, p-STS cannot play this role, since the association between seen and heard speech in deaf people is variable and relatively unsystematic. In the present study, not only was activation in this region observed in the absence of audition; it was *greater* in deaf than hearing people. One possibility is that activation by seen speech in p-STS is sensitive to the dominant speech modality within this multimodal region. That is, activation by silent speech in this region may be greater in deaf people because the region has developed to be sensitive to visual speech, while for hearing people it has developed to be sensitive to auditory speech characteristics, with visual speech as a secondary function. Structural imaging of the connections between p-STS and visual and auditory cortices in deaf and hearing individuals could be employed to test this hypothesis.

A non-mutually exclusive possibility is that greater activation in superior temporal regions for deaf than hearing individuals reflects a more general plasticity of these regions in deaf people. Several studies suggest that brain regions considered specialised for audition can be recruited for processing stimuli from other modalities in deaf people (e.g., Fine, Finney, Boynton, & Dobkins, 2005; Finney, Fine, & Dobkins, 2001; Sadato et al., 2005). While the extent and specificity of primary auditory cortex recruitment by visual events remains unclear (Bavelier, Dye, & Hauser, 2006), some studies (e.g., MacSweeney et al., 2004) suggest that perception of signed language, and even of non-linguistic biological movement, can recruit regions within superior temporal cortex to a greater extent in deaf native signers than in hearing people exposed to a signed language from birth (hearing native signers).

### 4.2 Correlations of activation with individual differences in speechreading skill

TAS speechreading scores and post-scan speechreading of the items seen in the scanner were positively correlated (deaf: *r* = 0.476, *p*(1-tailed) = 0.05; hearing: *r* = 0.673, *p*(1-tailed) = 0.034); thus we can infer that the higher the TAS score, the more likely it is that participants would have processed the speechread material lexically. However, TAS scores were not normally distributed across the two groups. For this reason, standard scores (TAS-*z*) derived for each group formed the basis for exploring the relationship between speechreading skill and cortical activation. Within each group, different patterns of association were observed. In deaf participants, the correlational analyses showed that activation in the posterior portion of the superior temporal gyri (as well as middle temporal and middle frontal gyri) was positively associated with speechreading.

In the hearing participants, who were less able and more varied speechreaders than the deaf participants, speechreading skill was positively associated with activation in the right lingual and posterior cingulate gyri, which is consistent with findings from Hall et al. (2005). Additional activations displaying a positive correlation with speechreading skill included the right postcentral and inferior temporal (fusiform) gyri, perhaps suggesting relatively greater involvement of articulatory skill and face processing in hearing individuals’ speechreading, respectively.

Taken together, these data show that hearing status is an important determinant of activation in left superior temporal regions when words are speechread. In particular, silent speechreading elicits greater activation in the left middle and posterior portions of the superior temporal cortex, including the superior and middle temporal gyri and the lateral portion of the transverse temporal gyrus in deaf than hearing people, even when speechreading skill is held constant. However, speechreading skill can moderate this activation, showing a positive relationship in deaf but not hearing participants. The relatively small group sizes used in the correlational analysis (*n* = 13 in each group), however, require that this interpretation should be provisional. Hall et al. (2005) did not find reliable activation in superior temporal gyrus for silent speechreading in contrast to viewing facial gurning in a group of 33 hearing participants, who also varied widely in speechreading skill. However, they did report a reliable positive correlation between speechreading skill and activation in this region. The inference from that study together with the present one must be that, when speechread material is linguistically processed, superior temporal regions within the left hemisphere are likely to be recruited. Additionally, the present study shows that it was deaf rather than hearing people who showed this relationship most clearly, and where individual differences in speechreading skill made an additional impact, despite the range of speechreading skill being larger in the hearing than the deaf group.

We have shown that, when auditory regions are not activated by acoustic stimulation, they can nevertheless be activated by silent speech in the form of speechreading. This finding may have some practical as well as theoretical significance. Current practice in relation to speech training for prelingually deaf children preparing for cochlear implantation emphasises acoustic processing. In auditory-verbal training, the speaking model is required to hide her or his lips with the aim of training the child's acoustic skills (e.g., Chan, Chan, Kwok, & Yu, 2000; Rhoades & Chisholm, 2000). Thus, a neurological hypothesis is being advanced which suggests that the deaf child should not watch spoken (or signed) language since this may adversely affect the sensitivity of auditory brain regions to acoustic activation following cochlear implantation. Such advice may not be warranted if speechreading activates auditory regions in both deaf and hearing individuals.

Speechreading gives access to spoken language structure by eye. It therefore has the potential to impact positively on the development of auditory speech processing following cochlear implantation. While there are few consistent correlates of improved post-implant speech processing in prelingually deaf cochlear implantees, efficiency in speechreading is implicated. For example, pre-implant silent speechreading skills are positively associated with general speech and language outcomes (Bergeson, Pisoni, & Davis, 2005). The possibility that superior temporal regions in deaf individuals, once tuned to visible speech, may then more readily adapt to perceiving speech multimodally should be seriously considered when recommendations concerning pediatric cochlear implantation procedures are being developed.

## Figures and Tables

**Table 1:** Participant characteristics: mean (S.D.) of age (in years), non-verbal IQ centile and speechreading (TAS) raw and *z*-scores

	Age	NVIQ centile	TAS	TAS *z*-score
Deaf (*n* = 13, 6 female)	27.4 (7.76) range: 18–49	88.2 (13.3) range: 50–98	32.54 (3.07) range: 27–37	0.436 (0.59) range: −0.62–1.29
Hearing (*n* = 13, 6 female)	29.4 (6.15) range: 18–43	83.2 (19.6) range: 25–99	25.08 (4.89) range: 15–34	0.183 (1.07) range: −2.02–2.14

**Table 2:** Activated regions for the perception of speech compared to baseline (static model) in deaf and hearing participants

	Hemisphere	Size (voxels)	*x*, *y*, *z*	BA
Deaf group				
Superior/middle temporal gyrus	R	246	51, −7, −3	22/21
Superior/transverse temporal gyrus	L	916	−54, −22, 10	42/41
Precentral gyrus	R	237	47, −4, 40	6
Medial frontal gyrus/anterior cingulate gyrus	L	211	−4, 15, 43	6/32

Hearing group				
Superior/middle temporal gyrus	R	451	43, −30, 0	22/21
Middle temporo-occipital junction	L	493	−43, −63, 0	37
Supramarginal gyrus	L	125	−33, −52, 43	40
Supramarginal gyrus	R	220	36, −48, 40	40
Precentral gyrus	L	457	−47, −7, 43	4/6
Inferior frontal gyrus	R	521	40, 11, 26	44
Medial frontal gyrus	R	218	4, 4, 50	6

Voxel-wise *p*-value = 0.05, cluster-wise *p*-value = 0.0025. Foci correspond to the most activated voxel in each 3-D cluster.

**Table 3:** Regions positively associated with speechreading skill (TAS *z*-scores) in deaf and hearing participants

	Hemisphere	Size (voxels)	*x*, *y*, *z*	BA
Deaf group				
Cerebellum	L	8	−11, −44, −30	–
Middle temporal gyrus	R	16	47, −26, −3	21
Insula	R	6	36, 15, 7	–
Transverse temporal gyrus	R	7	47, −19, 13	41
Superior temporal gyrus	L	16	−54, −26, 13	42
Dorsolateral prefrontal cortex	R	10	47, 22, 26	46
Anterior cingulate gyrus	–	7	0, 15, 33	32/24
Precentral gyrus	R	5	29, −7, 50	6/4
Middle frontal gyrus	L	6	−29, −4, 53	6
Middle frontal gyrus	R	6	33, 0, 53	6

Hearing group				
Fusiform gyrus	R	9	33, −44, −13	37
Lingual gyrus	R	8	11, −81, −7	18
Posterior cingulate gyrus	–	6	0, −33, 23	23
Posterior cingulate gyrus	L	5	−4, −11, 30	23
Postcentral gyrus	R	11	51, −15, 30	4

Voxel-wise *p*-value = 0.05, cluster-wise *p*-value = 0.0025. Foci correspond to the most activated voxel in each 3-D cluster.

## Footnotes

1 The modification of Friman et al. reflects the fact that, when combinations of gamma functions are used to model BOLD responses, some of the combinations that are possible mathematically are unlikely physiologically (i.e., they give rise to BOLD response shapes that are not observed in the brain). After analysing many thousands of BOLD curve shapes, Friman et al.'s solution was to suggest ranges of parameter combinations that were physiologically reasonable. In mathematical terms this consists of replacing the original gamma functions (*y*1(*t*) and *y*2(*t*)) by combinations (*y*1(*t*) + *ay*2(*t*) and *y*1(*t*) − *ay*2(*t*)) where *a* is a constant (0.3) derived from analysis of observed curve shapes.

2 The extent of activation in the left transverse temporal (i.e., Heschl's) gyrus for the speechreading condition as compared to the baseline condition was verified using the probability map provided by Penhune et al. (1996). Thirty-one voxels in the deaf group and four voxels in hearing group displayed ≥50% probability of being located within this region.

## References

[bib1] Bavelier D., Dye M.W., Hauser P.C. (2006). Do deaf individuals see better?. Trends in Cognitive Science.

[bib2] Bergeson T.R., Pisoni D.B., Davis R.A. (2005). Development of audiovisual comprehension skills in prelingually deaf children with cochlear implants. Ear and Hearing.

[bib3] Bernstein L.E., Auer E.T., Moore J.K., Ponton C.W., Don M., Singh M. (2002). Visual speech perception without primary auditory cortex activation. NeuroReport.

[bib4] Bernstein L.E., Demorest M.E., Tucker P.E. (2000). Speech perception without hearing. Perception & Psychophysics.

[bib5] Brammer M.J., Bullmore E.T., Simmons A., Williams S.C., Grasby P.M., Howard R.J. (1997). Generic brain activation mapping in functional magnetic resonance imaging: A nonparametric approach. Magnetic Resonance Imaging.

[bib6] Buccino G., Lui F., Canessa N., Patteri I., Lagravinese G., Benuzzi F. (2004). Neural circuits involved in the recognition of actions performed by nonconspecifics: An fMRI study. Journal of Cognitive Neuroscience.

[bib7] Bullmore E.T., Brammer M., Williams S.C., Rabe-Hesketh S., Janot N., David A. (1996). Statistical methods of estimation and inference for functional MR image analysis. Magnetic Resonance in Medicine.

[bib8] Bullmore E.T., Long C., Suckling J., Fadili J., Calvert G., Zelaya F. (2001). Colored noise and computational inference in neurophysiological (fMRI) time series analysis: Resampling methods in time and wavelet domains. Human Brain Mapping.

[bib9] Bullmore E.T., Suckling J., Overmeyer S., Rabe-Hesketh S., Taylor E., Brammer M.J. (1999). Global, voxel, and cluster tests, by theory and permutation, for a difference between two groups of structural MR images of the brain. IEEE Transactions on Medical Imaging.

[bib10] Calvert G.A., Brammer M.J., Bullmore E.T., Campbell R., Iversen S.D., David A.S. (1999). Response amplification in sensory-specific cortices during crossmodal binding. NeuroReport.

[bib11] Calvert G.A., Bullmore E.T., Brammer M.J., Campbell R., Williams S.C.R., McGuire P.K. (1997). Activation of auditory cortex during silent lipreading. Science.

[bib12] Calvert G.A., Campbell R., Brammer M.J. (2000). Evidence from functional magnetic resonance imaging of crossmodal binding in the human heteromodal cortex. Current Biology.

[bib13] Campbell R., MacSweeney M., Surguladze S., Calvert G., McGuire P., Suckling J. (2001). Cortical substrates for the perception of face actions: An fMRI study of the specificity of activation for seen speech and for meaningless lower-face acts (gurning). Brain Research. Cognitive Brain Research.

[bib14] Chan S.C., Chan S.K., Kwok I.C., Yu H.C. (2000). The speech and language rehabilitation program for pediatric cochlear implantees in Hong Kong. Advances in Oto-Rhino-Laryngology.

[bib15] Donoho D.L., Johnstone J.M. (1994). Ideal spatial adaptation by wavelet shrinkage. Biometrika.

[bib16] Edgington E.S. (1995).

[bib17] Fine I., Finney E.M., Boynton G.M., Dobkins K.R. (2005). Comparing the effects of auditory deprivation and sign language within the auditory and visual cortex. Journal of Cognitive Neuroscience.

[bib18] Finney E.M., Fine I., Dobkins K.R. (2001). Visual stimuli activate auditory cortex in the deaf. Nature Neuroscience.

[bib19] Friman O., Borga M., Lundberg P., Knutsson H. (2003). Adaptive analysis of fMRI data. Neuroimage.

[bib20] Friston K.J., Josephs O., Rees G., Turner R. (1998). Nonlinear event-related responses in fMRI. Magnetic Resonance in Medicine.

[bib21] Hall D.A., Fussell C., Summerfield A.Q. (2005). Reading fluent speech from talking faces: Typical brain networks and individual differences. Journal of Cognitive Neuroscience.

[bib22] Ludman C.N., Summerfield A.Q., Hall D., Elliott M., Foster J., Hykin J.L. (2000). Lip-reading ability and patterns of cortical activation studied using fMRI. British Journal of Audiology.

[bib23] MacSweeney M., Amaro E., Calvert G.A., Campbell R., David A.S., McGuire P. (2000). Silent speechreading in the absence of scanner noise: An event-related fMRI study. NeuroReport.

[bib24] MacSweeney M., Calvert G.A., Campbell R., McGuire P.K., David A.S., Williams S.C. (2002). Speechreading circuits in people born deaf. Neuropsychologia.

[bib25] MacSweeney M., Campbell R., Calvert G.A., McGuire P.K., David A.S., Suckling J. (2001). Dispersed activation in the left temporal cortex for speechreading in congenitally deaf speechreaders. Proceedings of the Royal Society of London B.

[bib26] MacSweeney M., Campbell R., Woll B., Giampietro V., David A.S., McGuire P.K. (2004). Dissociating linguistic and nonlinguistic gestural communication in the brain. Neuroimage.

[bib27] Mohammed T., Campbell R., MacSweeney M., Barry F., Coleman M. (2006). Speechreading and its association with reading among deaf, hearing and dyslexic individuals. Clinical Linguistics & Phonetics.

[bib28] Nishitani N., Hari R. (2002). Viewing lip forms: Cortical dynamics. Neuron.

[bib29] Paulesu E., Perani D., Blasi V., Silani G., Borghese N.A., De Giovanni U. (2003). A functional-anatomical model for lipreading. Journal of Neurophysiology.

[bib30] Pekkola J., Ojanen V., Autti T., Jaaskelainen I.P., Mottonen R., Tarkiainen A. (2005). Primary auditory cortex activation by visual speech: an fMRI study at 3 T. NeuroReport.

[bib31] Penhune V.B., Zatorre R.J., MacDonald J.D., Evans A.C. (1996). Interhemispheric anatomical differences in human primary auditory cortex: Probabilistic mapping and volume measurement from magnetic resonance scans. Cerebral Cortex.

[bib32] Rhoades E.A., Chisholm T.H. (2000). Global language progress with an auditory-verbal approach for children who are deaf or hard of hearing. Volta Review.

[bib33] Ruytjens L., Albers F., van Dijk P., Wit H., Willemsen A. (2006). Neural responses to silent lipreading in normal hearing male and female subjects. The European Journal of Neuroscience.

[bib34] Sadato N., Okada T., Honda M., Matsuki K., Yoshida M., Kashikura K. (2005). Cross-modal integration and plastic changes revealed by lip movement, random-dot motion and sign languages in the hearing and deaf. Cerebral Cortex.

[bib35] Scott S.K., Johnsrude I.S. (2003). The neuroanatomical and functional organization of speech perception. Trends in Neuroscience.

[bib36] Talairach, J., & Tournoux, P. (1988). *Co-planar stereotaxic atlas of the human brain* (M. Rayport, Trans.). New York: Thieme Medical Publishers, Inc.

[bib37] Watkins K.E., Strafella A.P., Paus T. (2003). Seeing and hearing speech excites the motor system involved in speech production. Neuropsychologia.

[bib38] Westbury C.F., Zatorre R.J., Evans A.C. (1999). Quantifying variability in the planum temporale: A probability map. Cerebral Cortex.

[bib39] Zeki S., Watson J.D., Lueck C.J., Friston K.J., Kennard C., Frackowiak R.S. (1991). A direct demonstration of functional specialization in human visual cortex. Journal of Neuroscience.

